# Multi-Feature Single Target Robust Tracking Fused with Particle Filter

**DOI:** 10.3390/s22051879

**Published:** 2022-02-27

**Authors:** Caihong Liu, Mayire Ibrayim, Askar Hamdulla

**Affiliations:** College of Information Science and Engineering, Xinjiang University, Urumqi 830046, China; caihongliu@stu.xju.edu.cn (C.L.); askar@xju.edu.cn (A.H.)

**Keywords:** adaptive learning rate update, adaptive filter update, correlation filtering, multi-feature fusion, particle filter re-detection

## Abstract

Aiming at the problems of target model drift or loss of target tracking caused by serious deformation, occlusion, fast motion, and out of view of the target in long-term moving target tracking in complex scenes, this paper presents a robust multi-feature single-target tracking algorithm based on a particle filter. The algorithm is based on the correlation filtering framework. First, to extract more accurate target appearance features, in addition to the manual features histogram of oriented gradient features and color histogram features, the depth features from the conv3–4, conv4–4 and conv5–4 convolutional layer outputs in VGGNet-19 are also fused. Secondly, this paper designs a re-detection module of a fusion particle filter for the problem of how to return to accurate tracking after the target tracking fails, so that the algorithm in this paper can maintain high robustness during long-term tracking. Finally, in the adaptive model update stage, the adaptive learning rate update and adaptive filter update are performed to improve the accuracy of target tracking. Extensive experiments are conducted on dataset OTB-2015, dataset OTB-2013, and dataset UAV123. The experimental results show that the proposed multi-feature single-target robust tracking algorithm with fused particle filtering can effectively solve the long-time target tracking problem in complex scenes, while showing more stable and accurate tracking performance.

## 1. Introduction

Computer vision [1] is a key technology for people to analyze and process visual images through the use of computers. The research on target tracking technology belongs to an important branch in the field of computer vision, which is one of the important means for humans and computers to transmit information to each other, and it is essential to the development of society. A large number of applications in video surveillance [2], shooting video [3], human–computer interaction [4], biological image analysis [5], military field [6], information security [7] and other fields show its important and unique status. Target tracking technology [8,9] integrates several fields such as image processing [10], pattern recognition and computer applications. Target tracking is the prediction of the size and position of the tracked target in the subsequent frames given the size and position of the target in the initial frame.

Compared with the traditional target tracking algorithm, the correlation filter target tracking algorithm appeared late, but because the correlation filter algorithm is relatively superior in tracking effect and efficiency, many domestic and foreign scholars introduced the correlation filter into the target tracking. To consider the variation of the tracking target over time and space, manual features such as color histogram features (CH) [11], directional gradient histogram features (HOG) [12], CN features [13], scale invariant features, and convolutional depth features extracted from deep networks such as VGGNet-16 [14] and VGGNet-19 [14] are introduced to represent the target object. Deep features are learned from a large number of training samples and are more discriminative than manual features, and thus have been widely used in target tracking in recent years. The team of Ma et al. [15] improved tracking accuracy and robustness by extracting the last three convolutional layer features and learning adaptive correlation filters on the deep convolutional neural network VGGNet-19 [14], pre-trained on ImageNet [16].

This paper studies the long-term target tracking problem in complex environments, especially the tracking problem of the target object under severe deformation, occlusion, fast motion, out of view, and so on. The HOG feature is a local feature, which can handle the minor morphological changes of the target and the problems caused by the illumination well. In complex environments where the background color and target color are similar, target tracking based on color histogram features (CH) alone is highly likely to lose the target. Depth features, as a feature with strong semantic information, are generally not disturbed by illumination or background color, so this paper fuses the feature response matrix of the three features of HOG features, CH features and depth features of images, and then trains a more robust appearance model.

In order to further improve the robustness and efficiency of long-term target tracking under the correlation filter architecture, a multi-feature single-target robust tracking algorithm fused with particle filter algorithm is proposed. The main contributions of this paper can be summarized as the following four points:

- First, the feature response matrix of three features, namely HOG features, CH features and depth features, is fused to train a more robust appearance model to improve the robustness of the algorithm in target tracking under complex environments such as severe target deformation, occlusion, fast motion, out-of-field, and similar colors;
- Second, we design a re-detection module incorporating particle filtering to address the problem of how to return to accurate tracking after the target tracking fails due to complex factors such as complete occlusion, deformation and blurring, so that the algorithm in this paper can maintain high robustness and efficiency when tracking for a long time;
- Third, in this paper, two parts, adaptive learning rate update and adaptive filter update, are performed in the adaptive model update phase to improve the robustness of the target model in this paper’s algorithm;
- Fourth, the proposed tracking algorithm is evaluated on the dataset OTB-2015, dataset OTB-2013 and dataset UAV123. and the experimental results show that the proposed algorithm exhibits more stable and accurate tracking performance in the case of severe target deformation, occlusion, fast motion, and out-of-field during tracking.

## 2. Related Work

### 2.1. Correlation Filtering

The first application of correlation filtering methods in the field of target tracking is in the MOSSE [17] algorithm proposed by Bolme et al., in 2010. The algorithm uses the correlation filter to learn the minimum output square error of the gray image of the target area. A measure of the similarity of candidate target regions to obtain the location of the tracking target in the image. In 2012, Henriques et al. proposed a detection-based kernel circulation structure tracking algorithm (CSK) [18], which improves the target tracking speed by using dense sampling to obtain samples, and then computes them using the fast Fourier transform. However, the CSK algorithm only extracts a single feature, and it cannot solve the problem of target scale changes [19].

In 2014, Henriques et al. proposed a high-speed tracking with kernelized correlation filters tracking algorithm KCF [20] in order to solve the single feature problem, which uses gradient histograms to extract features and enhances the ability of the algorithm to deal with target illumination and deformation. At the same time, it uses the cyclic matrix to collect samples to reduce the amount of calculations. In order to solve the problem of target scale change, Danelljan et al. proposed the DSST [21] algorithm in 2014. The DSST algorithm is based on the CSK/KCF algorithm framework, which first determines the target position through the position filter, and then adds a scale filter at the target position to calculate the scale with the largest response value to achieve scale adaptation. In the same year, Yang Li et al. proposed the scale adaptive multiple feature tracking algorithm SAMF [22]. The SAMF algorithm uses a multi-feature fusion method to improve tracking accuracy. At the same time, it uses a scale pool containing seven scales, and adjusts the scale adaptively through the magnitude of the scale change between the upper and lower frames.

In 2015, Danelljan et al. proposed a spatially regularized learning-based correlation filtering algorithm (SRDCF) [23], which mainly uses spatial regularization to reduce the response expansion search in the background region to improve the performance of the tracker. In 2015, Caloogahi et al. proposed correlation filters with limited boundaries (CFLB) [24] to address the boundary effect generated by spatial constraints in training the correlation filter. In order to make the algorithm more real-time, Ma et al. proposed a long-term tracking algorithm (LCT) [25] in 2015. The algorithm adds a detection mechanism and uses a classifier to redetect the target, which improves tracker processing power. In order to make the algorithm more sensitive to color changes, Bertinetto et al., in 2016 proposed a real-time tracking complementary learning tracking algorithm (Staple) [26], which adds color histogram features, solving the problem of deformation and lighting changes without affecting the tracking speed. In order to improve the tracking performance of the tracker while suppressing the boundary effect, in 2017, Lukezic et al. proposed a multi-channel and spatial reliability discrimination correlation filter (CSR-DCF) [27], which introduces channel and spatial reliability theory into the correlation filter, expands the region of interest and changes the shape of the tracking frame, and the robustness of the tracker is improved. In 2017, Caloogahi et al. proposed a background-aware correlation filter algorithm (BACF) [28], to address the drawback that the correlation filter cannot model the target over time solving the filter with ADMM optimization algorithm, reducing the amount of operations while improving the tracking accuracy of the tracker. In 2017, Matthiase et al. proposed a correlation filtering algorithm (CACF) [29] that combines global context and background information for training, which provides closed solutions for one-dimensional and multi-dimensional features in the original and dyadic domains, significantly improving the unstable tracking of the correlation filter with little impact on the tracking frame rate. In 2018, Cao et al. proposed an improved spatio-temporal context tracking algorithm based on spatio-temporal context fusion transfer learning theory [30,31], which effectively solved the location ambiguity problem in the tracker and improved the tracking performance of the tracker.

### 2.2. Tracking by Deep Neural Networks

In recent years, the deep convolution features extracted by deep neural networks have made significant progress in the application of computer vision. However, there are relatively few studies applying deep neural networks to visual tracking under the framework of correlation filtering architectures. One potential reason is that because the training data is very limited, the target position and scale are only available in the first frame. Fan et al. [31] learn a specific feature extractor with CNN from the offline training set for target tracking. Wang et al. [32] proposed a deep learning tracker DLT using a multilayer autoencoder network by pre-training it in an unsupervised manner. Wang et al. [33] learn video features by imposing temporal constraints. To alleviate the problem of offline training, DeepTrack [34] and CNT [35] methods learn target-specific CNNs step by step without pretraining. Existing deep network-based tracking methods [33,34,35] do not take full advantage of the rich hierarchical features but use two or fewer convolutional layers to represent the target objects.

**Figure 1:**
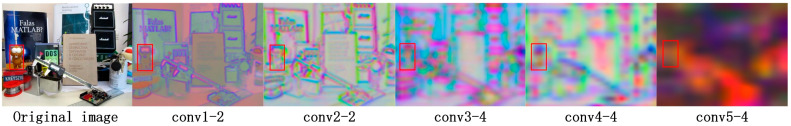
Visualization of the “lemming” image with features extracted on the five convolutional layers, conv1–2, conv2–2, conv3–4, conv4–4, and conv5–4, in VGGnet-19.

The algorithm in this paper extracts depth features using the VGGnet-19 [14] depth network trained on ImageNet [16] in a correlation filtering architecture. The fully connected layers of the VGGnet-19 [14] depth network are removed from the depth feature extraction process, and only the features extracted from the conv3–4, conv4–4, and conv5–4 convolutional layers are used. Figure 1 is a visual view of the depth features extracted from the “lemming” video image of the conv1–2, conv2–2, conv3–4, conv4–4, and conv5–4 five-layer convolutional layers of the VGGnet-19 [14] deep network.

## 3. The Proposed Method

**Figure 2:**
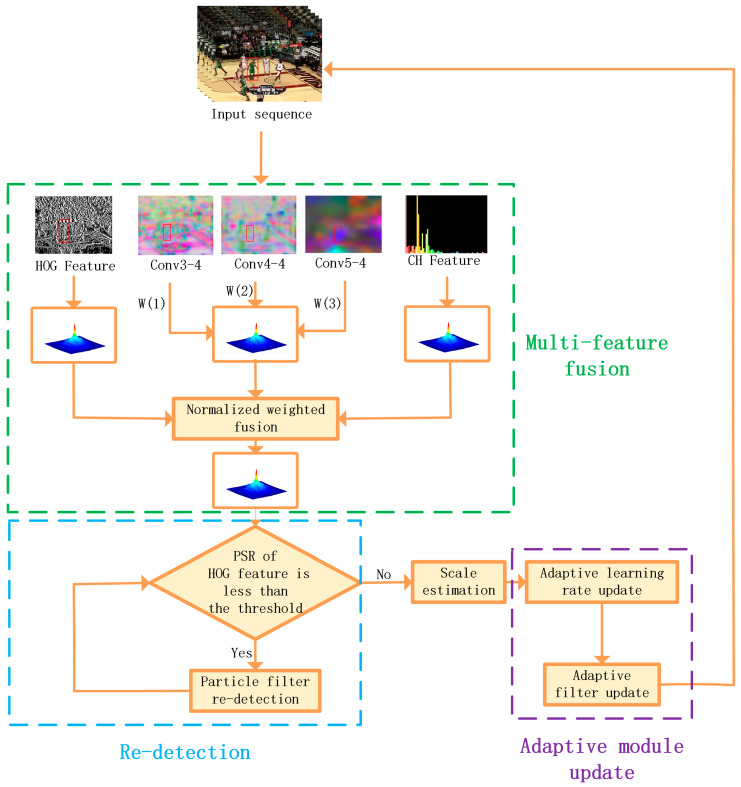
Framework diagram of the algorithm MFPF in this paper.

The multi-feature single-target robust tracking algorithm fused with particle filter algorithm in this article is based on the algorithm under the correlation filtering architecture, which will be described in Section 3.1. The response matrix of HOG features, CH features and depth features of the fused image during feature extraction is trained to produce a more robust appearance model, which will be explained in Section 3.2. Section 3.3 elaborates on the re-detection module of fused particle filtering designed for the problem of how to return to accurate tracking after target tracking failure. In the adaptive model update stage, the adaptive learning rate update and adaptive filter update will be introduced in detail in Section 3.4. The general framework of the algorithm proposed in this paper is shown in Figure 2. The proposed algorithm MFPF consists of three parts: multi-feature fusion, particle filter re-detection and adaptive module update, which are marked as green, blue, and purple boxes.

### 3.1. Correlation Filtering Architecture

To detect the tracked objects, the DCF model [17,20] is used to generate the response maps of the depth features and the HOG features, respectively, and the color histogram model [26,36] is used to generate the CH response maps. The three response maps are then scale normalized, hyperparameter weights are set, and weighted to generate the final response template, which determines the target location with the maximum response value.

The typical correlation filter tracker learns to discriminate the classifier and locates the target by searching the maximum value of the correlation response graph. The correlation filter-based tracker is trained using a block of images of size m×n. The training images are centered on the tracking target.

We consider all the circular shifts of x along the M and N dimensions as training samples. All the circular shifts of the patch $x_{m,n},\left(m,n\right)\in \left\{0,1,\ldots ,M-1\right\}\times \left\{0,1,\ldots ,N-1\right\}$ are generated as training samples with Gaussian function label $y\left(m,n\right)=e^{-\frac{{\left(m-M/2\right)}^{2}+{\left(n-N/2\right)}^{2}}{2\sigma^{2}}}$ in terms of the shifted distance, where σ is the kernel width. Then we learn the correlation filter w with the same size x by solving the following minimization problems:

(1) $$\begin{array}{c} min\\ f \end{array}\sum_{m,n}\|\emptyset \left(x_{m,n}\right)\cdot w-y{\left(m,n\right)\|}^{2}+\lambda \|w{\|}^{2}\;$$

where ∅ represents the mapping to a Hilbert space and λ is a regularization parameter (λ ≥ 0). By employing a kernel $k\left(x,x^{\prime }\right)=\langle \emptyset \left(x\right),\emptyset \left(x^{\prime }\right)\rangle$, the solution can be expressed as $w=\underset{m,n}{\sum }\alpha \left(m,n\right)k\left(x_{m,n},x\right)$, where α is the dual variable of w and it is defined by:

(2) $${\hat{\alpha }}^{*}=\frac{\hat{y}}{{\hat{k}}^{xx}+\lambda }\;$$

where $k^{xx}$ denotes the vector whose i-th element is $k\left(x_{i},x\right)$, the hat symbol denotes the discrete Fourier transform (DFT) of a vector (e.g., $\hat{\alpha }=F\left(\alpha \right)$) and ${\hat{\alpha }}^{*}$ is the complex-conjugate of $\hat{\alpha }$. The simplest linear kernel function is applied to our algorithm, $k\left(x,x^{\prime }\right)=g\left(x,x^{\prime }\right)$ for some function g, and $k^{xx^{\prime }}=g\left(F^{-1}\left(\hat{x}⨀{\hat{x}}^{\prime^{*}}\right)\right)$. In the tracking process, a patch z with the same size of x is cropped out in the new frame. The response map of z is calculated as follows:

(3) $$f\left(z\right)=F^{-1}\left({\left({\hat{k}}^{xz}\right)}^{*}⨀\;\hat{\alpha }\right)\;$$

where ⨀ is the element-wise product and *x* is the learned target appearance. To avoid the boundary effects during learning, we apply the Hann window to the signals. The online update is made as follows:

(4) $${\hat{x}}_{t}=\left(1-\mu_{h}\right){\hat{x}}_{t-1}+\mu_{h}{\hat{x}}_{t}^{\prime }\;$$

(5) $${\hat{\alpha }}_{t}=\left(1-\mu_{h}\right){\hat{\alpha }}_{t-1}+\mu_{h}{\hat{\alpha }}_{t}^{\prime }\;$$

where $\mu_{h}$ is the learning rate of HOG-based correlation filter and *t* is the index of the current frame. To avoid the contamination of the trained filter, $\mu_{h}$ in our framework is set adaptively. In this paper, two models are trained based on the correlation filter of one frame, one for position update and the other for scale update.

### 3.2. Multi-Feature Fusion

The manual features of the algorithm in this paper are composed of HOG features [10] and color histograms features [11]. The depth features use the last three convolutional layers conv3–4, conv4–4 and conv5–4 of the VGG19 network, with the weight of each layer set to 0.02, 0.5, and 1, respectively, similar to [15].

In the target tracking process, the manually calibrated position of the target is entered into the tracker at the moment of the first frame, the position information parameters are initialized in the tracker, and the target information described by each feature channel is modeled.

To facilitate the extraction of target information and background information, the target position is expanded and the search area is formed by 2.5 times the size of the target frame size. The correlation filter response matrix of HOG features and depth features are calculated by the template, and the integral image response matrix of CH features is calculated by Gaussian filtering, and then the response score matrix scales of the three features are normalized for feature information fusion to obtain the final response matrix. The fusion of each feature is calculated as shown in Equation (6):

(6) $$f\left(x\right)=\gamma_{hog}f_{hog}\left(x\right)+\gamma_{deep}f_{deep}\left(x\right)+\gamma_{ch}f_{ch}\left(x\right)\;$$

In Equation (6), $\gamma_{hog}$, $\gamma_{deep}$, and $\gamma_{ch}$ correspond to the hyperparameters of the respective feature information. Each correlation response represents the correlation result of the corresponding image block with the target template. Therefore, the final determination of the location of the target frame is done by finding the peak of the response score in the set of fused responses of each channel, and the location where the peak is located is the location where the target is defined by the algorithm in this paper. Table 1 shows the hyperparameter settings of the method in this paper.

After obtaining the target position, the target size is reduced using the scale pyramid expansion in the DSST [21] algorithm. The scale response score of each scale factor is calculated using the scale filter, and the scale factor at the peak of the scale response score is selected and set as the value of the target size change between the two frames.

### 3.3. Particle Filtering Re-Detection

In the redetection module, it is first discussed how to use the response of HOG features to estimate the reliability of tracking results. Then, particle filtering re-detection after target tracking failure is proposed. Particle filter re-detection is built on the basis of a correlation filter tracking algorithm apparatus, which evaluates the detection results for each frame. For the HOG-based correlation filter response map, the peak sidelobe ratio (PSR) can be calculated to quantify the sharpness of the correlation peak. If the PSR value is low, the correlation between the current frame and the previous frame is low. We defined the PSR of the correlation filter response map as the score $s_{h}^{\left(i\right)}$ of the HOG feature, which is calculated as in Equation (7), where $f_{h}^{\left(i\right)}$ is the ith response map of the HOG-based correlation filter, and $u_{i}$ and $\sigma_{i}$ are the mean and standard deviation of the response, respectively.

(7) $$s_{h}^{\left(i\right)}=\frac{max\left(f_{h}^{\left(i\right)}\right)-u_{i}}{\sigma_{i}}\;$$

The PSR values fluctuate somewhat for both position dependent and scale filters and can be much lower when unreliable tracking results occur. However, it is not appropriate to pre-define a constant threshold value to judge the reliability of the current tracking. Due to the uncertainty of tracking difficulty, the HOG scores of the response maps may fluctuate at different values. For example, when the PSR is below some set threshold, it means that the target tracking fails in video A, while it may indicate successful target tracking in video B due to the inclusion of more challenging factors in video B. Therefore, this paper considers the average score of each frame of the video to estimate the reliability of tracking results. We computed the HOG scores for each frame and combined them into a set $C_{h}=\left\{s_{h}^{\left(1\right)},s_{h}^{\left(2\right)},\ldots ,s_{h}^{\left(i\right)}\right\}$. We define $M_{h}$ as the average of the overall $C_{h}$. In this paper, a reliable fraction $S_{h}$ is selected into the set $C_{h}$ by defining a factor A for $M_{h}$. If $s_{h}^{\left(i\right)}<A\cdot M_{h}$, which usually implies some degree of occlusion or deformation, the result of the i th frame is considered to perform poorly and the corresponding HOG score is discarded, while a particle filtering re-detection is performed to re-track the target. Conversely, it means that the target tracking is not lost, and the result of that frame is integrated into the set $C_{h}$, and passes the new target position information to the tracker.

The idea of particle filtering is a statistical method based on Bayesian estimation [37] and Monte Carlo methods [38], by finding a set of random samples propagating in the state space to approximate the probability density function, replacing the integration operation with the sample mean, and then obtaining the process of minimum variance estimation of the system state. The selection of candidate objects by the particle filtering method can be regarded as a discrete sampling process, while the response map calculated by using correlation filtering can search for the best target location from a dense candidate region.

In the tracking process, if the tracking results are inaccurate and the target needs to be re-detected, draw N candidates around the tracked result in the previous frame using a particle filter. For each candidate x, it is represented by template set $D=\left[D_{+}\;D_{-}\right]$ with coefficients $\alpha =\left[\alpha_{+}\;\alpha_{-}\right]$, which is obtained by Equation (8):

(8) ‖αminx−Dα‖22+λ‖α‖1

where template set D contains $N_{p}$ positive templates $D_{+}$ near the object (e.g., within a radius of a few pixels) and $N_{n}$ negative templates $D_{-}$ far away from the object. A candidate with smaller reconstruction error using positive template set $D_{+}$ is more likely to be a target and vice versa. Thus, by computing the reconstruction error of each candidate using template D, we can predict the reliability $R_{i}$ of the *i*-th candidate roughly:

(9) $$R_{i}=\|x_{i}-D_{-}\alpha_{-}{\|}_{2}^{2}-\|x_{i}-D_{+}\alpha_{+}{\|}_{2}^{2}$$

where $\|x_{i}-D_{-}\alpha_{-}{\|}_{2}^{2}$ is the reconstruction error using negative template set $D_{-}$ and $\alpha_{-}$ is the corresponding coefficient vector, and $\|x_{i}-D_{+}\alpha_{+}{\|}_{2}^{2}$ is computed in a similar way. For the i-th candidate, higher $R_{i}$ means its higher possibility of a target. Although this method is not robust enough to redetect the target, it provides holistic information of the target and we can discard many useless candidates for efficiency. In our algorithm, we discard 90% of the candidates through reconstruction error, which predicts the location roughly and reduces the computational cost greatly in the accurate location process. The rest of the candidates will be exploited by particle filter for accurate target localization as discussed in the following.

We compute the combined response map using Equation (3) for the selected candidates and the final confidence score $S_{i}$ is defined as follows:

(10) $$S_{i}=max\left(f_{i}\right)\times cos\left(\frac{\beta }{W_{t}+H_{t}}\|L_{p}^{i}-L_{t}\|\right)$$

In the above equation, $f_{i}$ is the correlation response matrix corresponding to the i-th candidate. cos(·) corresponds to the distance score, where $W_{t}$ and $H_{t}$ are the width and height of the target position at the moment of frame t. β is a predefined distance penalty parameter, and the value of β is set to π/9. $L_{p}^{i}$ and $L_{t}$ are the target center position of the corresponding particle sample and the target center position before the deviation of the target position. Similar to the tracking detection part, the position of the redetected target is determined by searching the maximum value of the best candidate response map $S_{i}$. Then the threshold value of $S_{h}$ is judged. A value greater than the threshold value indicates that the target is not lost, and a value less than the threshold value indicates that the target tracking is deviated, then the particle filtering re-detection is needed again until the target is accurately tracked.

The proposal of the particle filter re-detection module, on the one hand, can avoid the special situation of poor tracking effect affecting the subsequent target tracking, and improve the performance of the algorithm. On the other hand, in the process of target tracking, if the target tracking is normal, the re-detection module is not activated, which greatly reduces the computational complexity.

### 3.4. Adaptive Update

#### 3.4.1. Adaptive Learning Rate Update

In the target tracking process, the tracking method with fixed model update rate has poor tracking effect in scenes with target deformation and occlusion. Target deformation causes the target’s appearance characteristics to change significantly between consecutive frames, and the tracking method with fixed model update rate generally uses a smaller model update rate in order to achieve better results on the overall data set, so the tracking model update soon lags behind the target appearance changes, eventually leading to tracking drift. Similarly, in the occlusion scene, when the target is occluded by the background object, the fixed model update rate method will continue to update the tracking model, resulting in the target model being contaminated by the background occlusion and eventually leading to tracking failure, so adaptive learning rate update is considered in the tracking process.

In the tracking process, the target model learning rate $\mu_{h}$ is reduced when the tracked target is considered unreliable to avoid the update of target changes due to severe target deformation, occlusion, fast motion, etc., and to reduce the possibility of target model update errors. Considering that the correlation filter learns target information and background information per frame, the feature information of each frame is fully utilized by the designed Equation (11):

(11) $$\mu_{h}=\{\begin{array}{c} P\\ v{\left(S_{h}/M_{h}\right)}^{\gamma }P\;\;\;\;\;\;\; \end{array}$$

where P is a constant, γ is the power exponent of the power function, and v∈[0,1] is the penalty factor that limits the maximum learning rate. The power function is designed to maintain reliable samples and severely penalize samples with low scores.

#### 3.4.2. Adaptive Filter Update

Most existing trackers do not consider whether the detection is accurate or not. In fact, once the target is detected incorrectly in the current frame, severe occlusion or complete loss can lead to tracking failure in subsequent frames. In this paper, in addition to the maximum response value $F_{max}$ of the reference response map, we also add the average peak correlation energy criterion. The detection result in the current frame can only be considered as high confidence when both criteria $F_{max}$ and APCE of the current frame are greater than their respective historical averages by a certain ratio, indicating that there is no tracking failure of the target, and then the learning rate is updated according to the model. The average peak correlation energy criterion is defined as follows:

(12) $$APCE=\frac{{\left|F_{max}-F_{min}\right|}^{2}}{mean\left(\sum_{w,h}{\left(F_{w,h}-F_{min}\right)}^{2}\right)}$$

where $F_{max}$ and $F_{min}$ are the maximum and minimum responses of the current frame, respectively. $F_{w,h}$ is the element value of the wth row and hth column of the response matrix. Adding the APCE standard to the model update strategy alleviates the target model offset problem to a certain extent and thus improves the accuracy of target tracking. Algorithm 1 describes the algorithmic process of this paper, where APCE_Average is the average of all the APCE values in the calculated video sequence frames divided by the number of frames; $F_{max}$ Average is the average of all the response values in the calculated video sequence frames divided by the number of frames.
**Algorithm 1:** Multi-Feature Single-Target Robust Tracking Algorithm Fused with Particle Filter**Input:** initial target position $\left(X_{i-1},Y_{i-1},W_{i-1},H_{i-1}\right)$ and other initialization parameters
**Output:** estimated target location $\left(X_{i},Y_{i},W,H_{i},\right)$
1.     Enter the first frame and initialize the target filter model;
2.     **for** i = 2,3,…, until the last frame **do**
3.          Determine the search window in the i-th frame;
4.            Extract the HOG, CH and depth features and calculate the corresponding correlation response maps;
5.          Feature fusion using (6);
6.          Use (6) to calculate the score of the current target $s_{h}^{\left(i\right)}$;
7.         **if** $s_{h}^{\left(i\right)}<A\cdot M_{h}$ **then**
8.           Particle filtering re-detection for the current frame target;
9.           Use Equation (10) to calculate the confidence of the candidate;
10.             Choose the best candidate $S_{i}$;
11.         **end if**
12.        Determine the optimal scale of the target and calculate the scale filter;
13.        Use (11) to adaptively update the learning rate $\mu_{h}$;
14.        Use (12) to calculate APCE value;
15.         **if** APCE > 0.45 * APCE_Average and $F_{max}$ > 0.6 * $F_{max\_}$ Average **then**
16.           Use (4) and (5) to update the filter;
17.         **end if**
18.       **end for**

## 4. Implementation Details

To verify the performance of the algorithm, the imagenet-vgg-verydeep-19.mat model is used and experiments are performed under the convolutional neural network toolbox matconvnet. In this paper, we use Matlab2018a programming and the hardware environment is Intel(R) HD Graphics 630. VGGNet-19 [14] trained on the ImageNet [16] dataset is used as the feature extractor of the algorithm in this paper. The depth features use the last three convolutional layers conv3–4, conv4–4, and conv5–4 of the VGGNet-19 [14] network, and to remove the boundary discontinuities, the extracted feature channels of each convolutional layer were weighted by a cosine frame filter, with the weights of each layer set to 0.02, 0.5, and 1, respectively, similar to [15]. In Equation (6), $\gamma_{hog}$, $\gamma_{deep}$, and $\gamma_{ch}$ corresponding to the hyperparameters of the respective feature information are set to 0.3, 0.3, and 0.4, respectively. The threshold A in the redetection is set to 0.6, and in the update of the adaptive learning rate, P in Equation (11) is set to 0.01, the penalty factor v is set to 0.8, and the power exponent γ is set to 3.

## 5. Experiment Results and Analysis

The OTB-2015 dataset [39] has a total of 100 video sequences, the OTB-2013 dataset [40] has a total of 51 video sequences, and the UAV123 dataset [6] has a total of 123 video sequences. Both OTB-2015 dataset and OTB-2013 dataset include 11 scene challenges, namely illumination change (IV), scale change (SV), occlusion (OCC), deformation (DEF), motion blur (MB), fast motion (FM), in-plane rotation (IPR), out-of-plane rotation (OPR), out of view (OV), background clutter (BC), and low resolution (LR). The UAV123 dataset contains 12 challenge scenarios, namely illumination change (IV), scale change (SV), fast motion (FM), background clutter (BC), low resolution (LR), full occlusion (FOC), partial occlusion (POC), out of view (OV), similar object (SOB), aspect ratio change (ARC), camera motion (CM), and viewpoint change (VC). In order to compare the performance of each algorithm, two metrics are used to evaluate the algorithm in this paper.

The first metric is precision. Precision is defined as the percentage of the total number of frames in the video sequence for which the difference between the center position of the tracking and the standard center position is less than a certain threshold. The percentage obtained varies by setting different thresholds, and the threshold is set to 20 in this experiment. The second metric is the success rate. The success rate is defined as the ratio of the area of the overlapping part of the tracking frame and the standard frame to the total area covered in the current frame, which is the value of VOR. We consider the tracking successful if the obtained VOR value exceeds a specific threshold; the VOR value is set to 50 in this experiment.

### 5.1. Experiments on the OTB2015

#### 5.1.1. Quantitative Analysis

**Figure 3:**
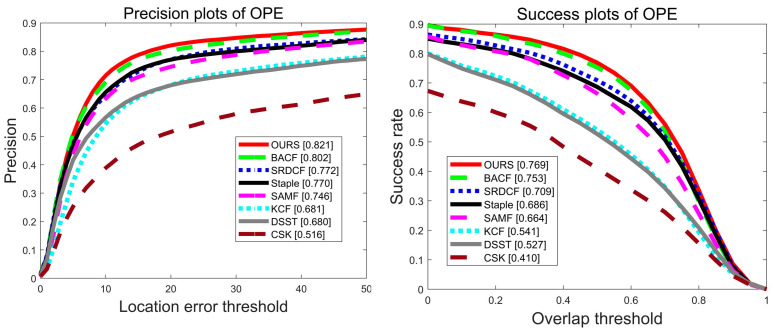
Precision plot and success plot of the eight algorithms on the OTB-2015 dataset.

Experiments are performed on 100 video sequences of the OTB2015 dataset, and Figure 3 shows the precision plot and success plot of eight algorithms, CSK [17], DSST [21], KCF [20], SAMF [22], Staple [26], SRDCF [23], BACF [30], and OURS on the OTB-2015 dataset. Compared with the benchmark algorithm Staple [26], the algorithm in this paper has improved 5.1% and 8.3% in accuracy and success rate, respectively, as shown by the red line. The comparison with other trackers on the OTB-2015 dataset in terms of accuracy metrics and success metrics is shown in Table 2. Red font represents the best performance, blue font indicates the second best, and green font indicates the third.

**Figure 4:**
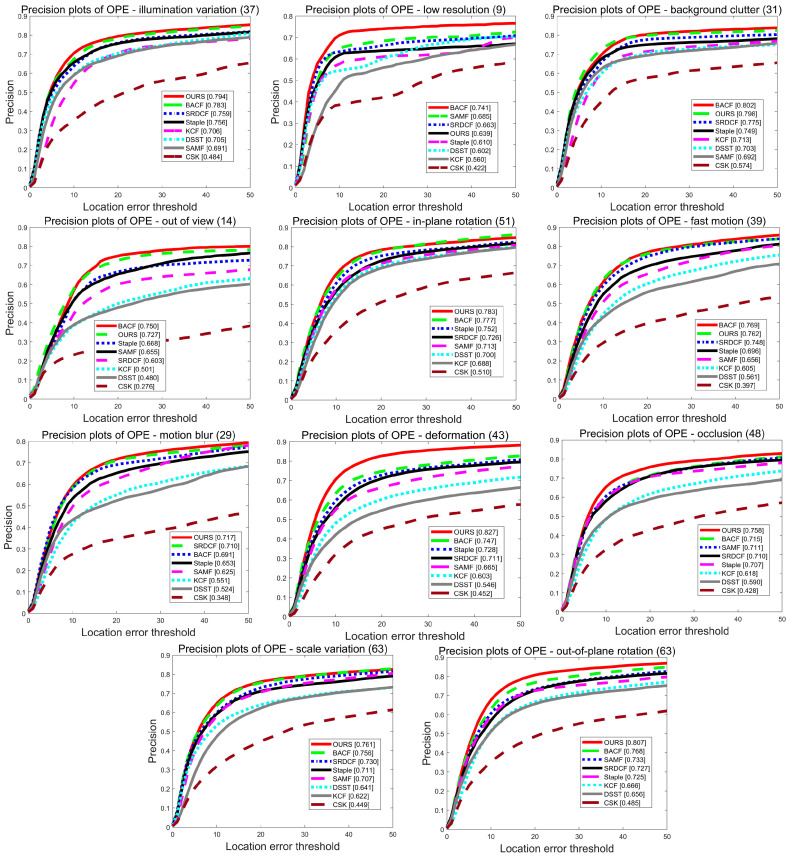
The precision plot of eight algorithms for 11 challenge scenarios on the OTB-2015 dataset.

**Figure 5:**
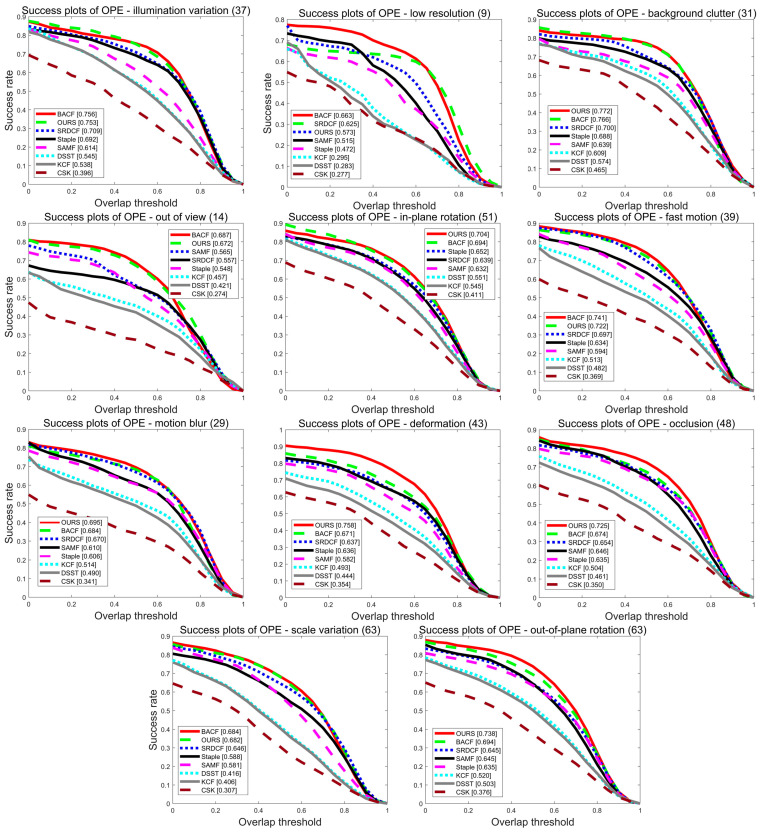
The success plot of eight algorithms for 11 challenge scenarios on the OTB-2015 dataset.

To further analyze the tracking performance of the algorithms in this paper, the advantages of the algorithms in this paper were also demonstrated by comparing 11 challenge scenarios in the OTB-2015 dataset sequence. The analysis with the eight algorithms based on the 11 challenge scenarios is shown in Figure 4 and Figure 5, with the challenge scenarios displayed in the title of each graph and the number of videos appended to the end of each title. In this paper, the MFPF algorithm achieves the best performance in multiple scenarios for accuracy metrics and success metrics on all 11 challenge scenarios, respectively. In the success rate graph for the out of view scenario, the algorithm in this paper outperforms the Staple algorithm by 9.9% in terms of score. In the target deformation scenario, the Staple algorithm has an AUC score of 54.8%. The tracker in this paper again provides an improvement of 12.4% compared to the Staple algorithm.

#### 5.1.2. Qualitative Analysis

**Figure 6:**
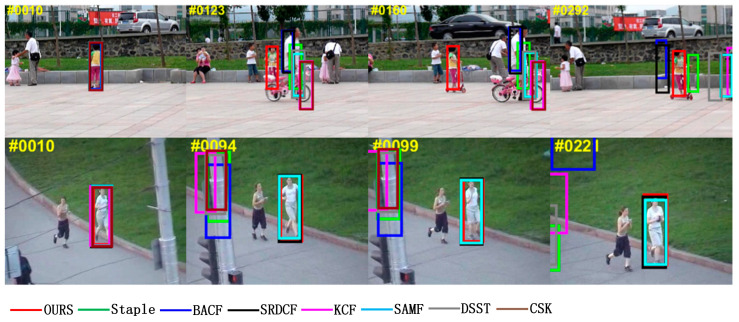
Visual comparison with eight trackers in the “Girl2” and “Jogging2” video sequences in an occlusion challenge scenario.

Occlusion can contaminate the target model and will lead to irreversible errors if no measures are taken to eliminate this interference. The “Girl2” video sequence in Figure 6 shows the case where the target suffers from short-term complete occlusion, and among these compared algorithms, only the MFPF algorithm in this paper can handle it and achieve accurate tracking of the target, as shown in the red borders in frames 123, 160, and 292 of the “Girl2” video sequence, shown in Figure 6. The “Jogging2” video sequence shows the target suffering from short-term partial occlusion, and only algorithms SRDCF, SAMF and MFPF of this paper are successful, as shown in Figure 6 for frame 94, frame 99, and frame 221 of the “Jogging2” video sequence with black, blue and red borders. The successful tracking of SRDCF is attributed to the spatial regularization to improve the tracking robustness, the successful tracking of SAMF is attributed to the use of multi-features and scale filters, and the successful tracking of MFPF is attributed to the multi-features fusion to enhance the feature information representation and the particle filtering to re-detect after tracking failure.

**Figure 7:**
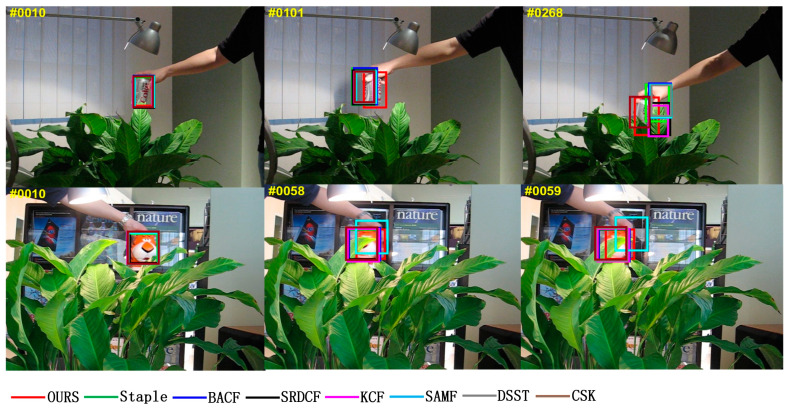
Visual comparison with eight trackers in the “Coke” and “Tiger2” video sequences in an occlusion challenge scenario.

In the video sequence of “Coke” in Figure 7, the light intensity is normal at frame 10, and only the algorithm in this paper can accurately track the target by increasing the light intensity at frames 101 and 268. Similarly, in the “Tiger2” video sequence, the light intensity is normal at frame 10, and only the algorithm in this paper can accurately track the target by increasing the light intensity at frames 58 and 59. In the tracking process, compared with the other seven algorithms, the algorithm MFPF in this paper has the highest accuracy in tracking the target in the case of large luminance changes. Attributed to the multi-feature fusion to enhance feature information expression and adaptive update learning rate, the trained filter is more robust and has a better tracking effect.

**Figure 8:**
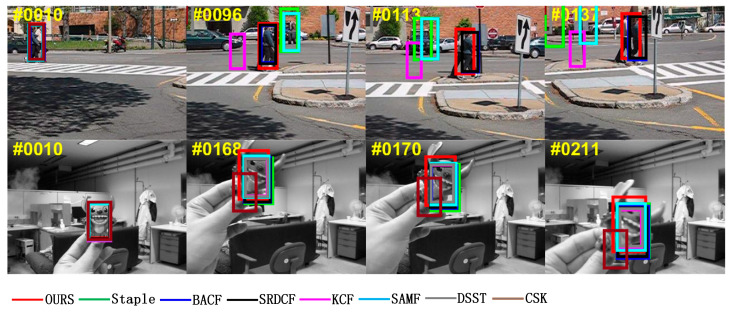
Visual comparison with eight trackers in the “Couple_1” and “Toy_1” video sequences in an occlusion challenge scenario.

In the deformation from frame 96 to frame 127 of the video sequence “Couple_1” in Figure 8, the SRDCF algorithm and the MFPF algorithm in this paper are able to locate and track the target accurately even if all the other six trackers fail to track, shown in the black and red box. In the frames 168, 170 and 211 of the “Toy_1” video sequence, only the MFPF algorithm in this paper is the most accurate in tracking the target deformation, shown in the red box, which is attributed to the use of particle filtering re-detection after the tracking failure of the MFPF algorithm, which enhances the robustness of the target tracking model.

**Figure 9:**
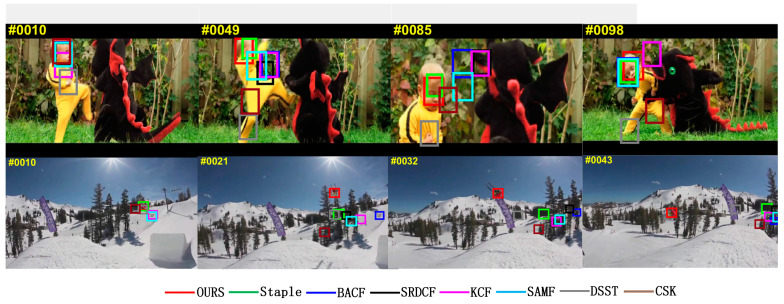
Visual comparison with eight trackers in the “DragonBaby_1” and “Skiing_1” video sequences in an occlusion challenge scenario.

Fast motion blurs the target and we need a wider search range to ensure that the target can be captured again. The video sequence in Figure 9 is used to test the performance of the tracker in handling fast moving targets, and only the Staple algorithm and MFPF algorithm can track the target in the “DragonBaby_1” video sequence, shown in the green and red box. The “Skiing_1” video sequence has only the MFPF algorithm that can track the target when fast motion occurs, as shown in the red borders in frames 21, 32, and 43 of the “Skiing_1” video sequence, shown in Figure 9. The reason for the high robustness of the MFPF algorithm is that it uses a search window of 2.5 times the size of the target and an adaptive update strategy, which ensures that the target is not easily lost when moving fast.

**Figure 10:**
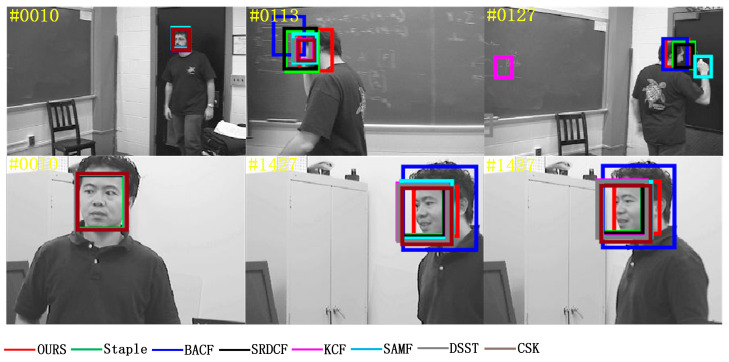
Visual comparison with eight trackers in the “Freeman1_1” and “Mhyang_1” video sequences in an occlusion challenge scenario.

Rotation is caused by the movement or change in viewpoint of the target, a challenge that makes it difficult to model the appearance of the target. In the rotation tests of the “Freeman1_1” and “Mhyang_1” video sequences in Figure 10, most trackers were able to track the target, but some trackers experienced significant scale drift due to the rotation of the target in the image plane. In the “Mhyang_1” video sequence, only the algorithm MFPF in this paper can closely track the target and maintain a high degree of overlap in frames 427 and 437, as shown in the red box, which indicates that the MFPF algorithm can well solve the challenge of in-plane rotation of the target.

**Figure 11:**
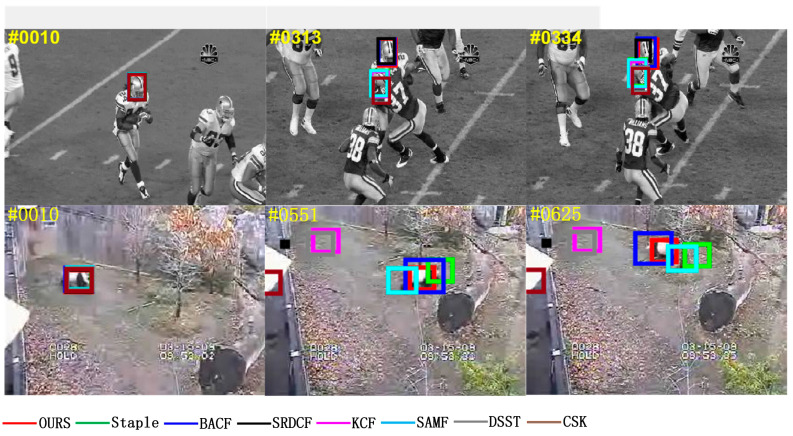
Visual comparison with eight trackers in the “Football_1” and “Panda_1” video sequences in an occlusion challenge scenario.

The background clutter is a challenge in the “Football_1” and “Panda_1” video sequences in Figure 11, which caused the bounding box to drift into the background. As seen in frames 113 and 127 of the “Football_1” video sequence and frames 551 and 652 of the “Panda_1” video sequence, only the MFPF algorithm proposed in this paper is always able to track the target accurately, as shown in the red box. It is very difficult to distinguish the target object from the background by traditional models, and the MFPF algorithm has the advantage of using more robust feature information and adaptive learning rates and filter updates to train a more robust tracker, which in turn leads to more efficient and accurate tracking.

### 5.2. Experiments on the OTB2013

#### 5.2.1. Quantitative Analysis

**Figure 12:**
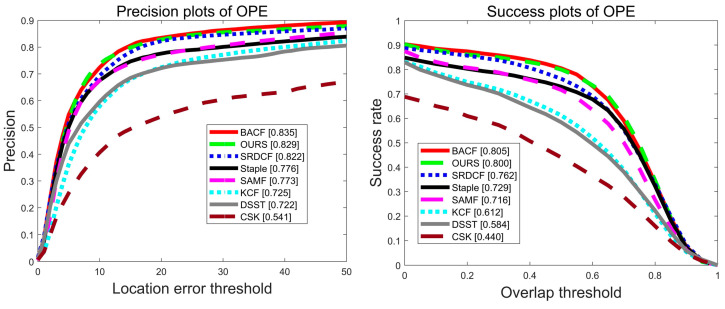
Precision plot and success plot of eight algorithms on the OTB-2013 dataset.

Experiments are conducted on 51 video sequences of the OTB2013 dataset, and Figure 12 shows the precision plot and success plot of eight algorithms, CSK [17], DSST [21], KCF [20], SAMF [22], Staple [26], SRDCF [23], BACF [30], and OURS on the OTB-2015 dataset. Compared with the benchmark algorithm Staple [26], the algorithm MFPF in this paper improves precision and success rate by 5.3% and 7.1%, respectively. The comparison with other trackers on the OTB-2013 dataset in terms of accuracy metrics and success metrics is shown in Table 3. Red font represents the best performance, blue font indicates the second best, and green font indicates the third.

#### 5.2.2. Ablation Experiment

**Figure 13:**
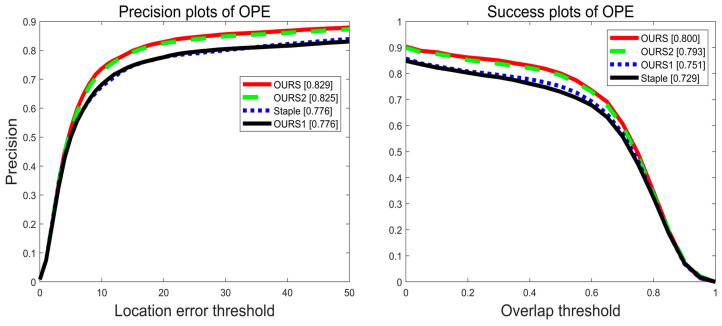
Precision plot and success plot of four algorithms on the OTB-2013 dataset.

The algorithm in this paper is based on the Staple tracker that performs multi-feature fusion of HOG features, depth features, and CH features in the feature information extraction stage, and accurately track the target again by using particle filtering for target re-detection after target tracking failure. Adaptive learning rate update and adaptive filter update are performed in the model update phase to make the trained filter more robust and enable better tracking. Figure 13 shows the ablation experiment of the algorithm in this paper on the OTB-2013 dataset. The OURS1 algorithm is a multi-feature fusion with HOG features, depth features, and CH features added to the benchmark algorithm Staple. The OURS2 algorithm is a particle filtering re-detection module after target tracking failure was added to the OURS1 algorithm. The OURS algorithm has an adaptive learning rate update and an adaptive filter update added.

The OURS1 algorithm does not improve in tracking accuracy compared to the benchmark algorithm Staple, and only improves in success rate by 2.2%. The reason for this situation may be that adding depth features creates feature redundancy when extracting feature information in some tracking sequences, resulting in no change in tracking accuracy. OURS2 algorithm combines particle filtering re-detection on the basis of OURS1 algorithm and improves in tracking accuracy by 4.9% and tracking success rate by 4.2%. The OURS algorithm incorporates adaptive learning rate and filter update based on the OURS2 algorithm, which improves tracking accuracy by 0.4% and tracking success rate by 0.7% compared to the OURS2 algorithm.

### 5.3. Experiments on the UAV123

**Figure 14:**
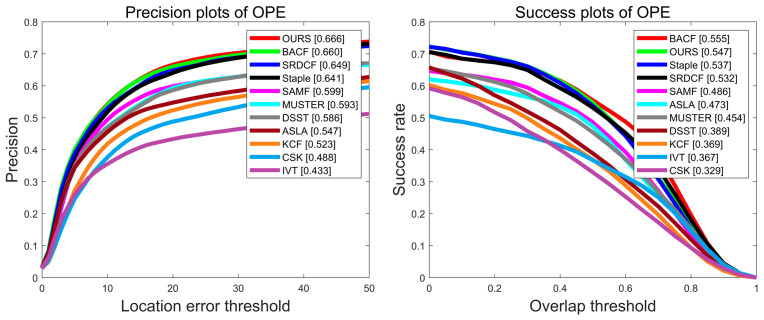
Precision plot and success plot of eleven algorithms on the UAV123 dataset.

The UAV123 dataset contains 123 video sequences taken by an unmanned aerial vehicle (UAV), including search and rescue, wildlife and crowd monitoring, navigation, etc. The average sequence length of this dataset is 915 frames. It contains a large number of long-term video tracking sequences, which present great difficulty and challenge to the trackers. Especially for trackers without a relocation mechanism, once model drift occurs, tracking fails. Figure 14 shows the precision plot and success plot of eleven algorithms, CSK [17], DSST [21], KCF [20], SAMF [22], Staple [26], SRDCF [23], BACF [30], ASLA [41], IVT [42], MUSTER [43], and OURS on the UAV123 dataset. Compared with the benchmark algorithm Staple, the algorithm MFPF in this paper improves the accuracy and success rate by 2.5% and 1%, respectively. The comparison with other trackers on the UAV123 dataset in terms of accuracy metrics and success metrics is shown in Table 4. Red font represents the best performance, blue font indicates the second best, and green font indicates the third.

## 6. Conclusions

In this paper, a multi-feature single target robust tracking fused with particle filter based on the correlation filtering framework is proposed. In the feature extraction stage, fusing manual features and depth features, the stronger the feature information extracted from the tracking target, the more accurate the trained filter tracking effect is. During the target tracking process, when the calculated PSR value is lower than the set threshold, the target shows tracking inaccuracy information, the target is re-tracked and re-located using particle filtering re-detection.

In the model update phase, two parts, adaptive learning rate update and adaptive filter update, are performed. The adaptive learning rate update is reflected in the tracking process, when the tracking target is considered unreliable, the target model learning rate is reduced to avoid the update of target changes due to severe target deformation, occlusion, and fast motion, and to reduce the possibility of target model update errors. The adaptive filter update is reflected in locating the target position. In addition to referring to the maximum response value of the response map, the average peak correlation energy APCE criterion is also added. The addition of the APCE criterion in the model update strategy alleviates the target model offset problem to a certain extent, thereby improving the target tracking accuracy.

The experimental results show that the proposed tracker performs well in terms of tracking precision and success rate compared to several state-of-the-art algorithms on the OTB-2013 dataset, the OTB-2015 dataset, and the UAV123 dataset. However, the algorithm is computationally complex due to the use of depth features, which does not achieve the purpose of real-time tracking, and further research is needed to enhance the speed of this algorithm.

## Figures and Tables

**Table 1:** Multi-feature fusion hyperparameter settings.

$\gamma_{hog}$	$\gamma_{deep}$	$\gamma_{ch}$
0.3	0.3	0.4

**Table 2:** Average performance of algorithms on the OTB-2015 dataset.

Tracker	Precision	Success	Average Scores
OURS	82.1%	76.9%	79.5%
Staple	77.0%	68.6%	72.8%
BACF	80.2%	75.3%	77.8%
SRDCF	77.2%	70.9%	74.1%
SAMF	74.6%	66.4%	70.5%
KCF	68.1%	54.1%	61.1%
DSST	68.0%	52.7%	60.4%
CSK	51.6%	41.0%	46.3%

**Table 3:** Average performance of algorithms on the OTB-2013 dataset.

Tracker	Precision	Success	Average Scores
OURS	82.9%	80.0%	81.5%
Staple	77.6%	72.9%	75.3%
BACF	83.5%	80.5%	82.0%
SRDCF	82.2%	76.2%	79.3%
SAMF	77.3%	71.6%	74.5%
KCF	72.5%	61.2%	66.9%
DSST	72.2%	58.4%	65.3%
CSK	54.1%	44.0%	49.1%

**Table 4:** Average performance of algorithms on the UAV123 dataset.

Tracker	Precision	Success	Average Scores
OURS	66.6%	54.7%	60.7%
Staple	64.1%	53.7%	58.9%
BACF	66.0%	55.5%	60.8%
SRDCF	64.9%	53.2%	59.1%
SAMF	59.9%	48.6%	54.3%
KCF	52.3%	36.9%	44.6%
DSST	58.6%	38.9%	48.8%
CSK	48.8%	32.2%	40.5%
MUSTER	59.3%	45.4%	52.4%
ASLA	54.7%	47.3%	51.0%
TVT	43.3%	36.7%	40.0%

## Data Availability

Publicly available datasets were analyzed in this study. This data can be found here: http://cvlab.hanyang.ac.kr/tracker_benchmark/datasets.html (accessed on 5 January 2022).

## References

[1] Janai J., Güney F., Behl A., Geiger A. (2020). Computer vision for autonomous vehicles: Problems, datasets and state of the art. Foundations and Trends® in Computer Graphics and Vision.

[2] Ruan W., Chen J., Wu Y., Wang J., Liang C., Hu R., Jiang J. (2018). Multi-correlation filters with triangle-structure constraints for object tracking. IEEE Trans. Multimed..

[3] Previtali F., Bloisi D.D., Iocchi L. (2017). A distributed approach for real-time multi-camera multiple object tracking. Mach. Vis. Appl..

[4] Rautaray S.S., Agrawal A. (2015). Vision based hand gesture recognition for human computer interaction: A survey. Artif. Intell. Rev..

[5] Liu Y., Jing X.-Y., Nie J., Gao H., Liu J., Jiang G.-P. (2018). Context-aware three-dimensional mean-shift with occlusion handling for robust object tracking in RGB-D videos. IEEE Trans. Multimed..

[6] Mueller M., Smith N., Ghanem B. (2016). A benchmark and simulator for UAV tracking. Computer Vision—ECCV 2016, Proceedings of the European Conference on Computer Vision, Amsterdam, The Netherlands, 11–14 October 2016.

[7] Wang T., Zhang G., Bhuiyan M.Z.A., Liu A., Jia W., Xie M. (2020). A novel trust mechanism based on fog computing in sensor-cloud system. Future Gener. Comput. Syst..

[8] Meng L., Yang X. (2019). A survey of object tracking algorithms. Acta Autom. Sin..

[9] Li P., Wang D., Wang L., Lu H. (2018). Deep visual tracking: Review and experimental comparison. Pattern Recognit..

[10] Wang X., Wang G., Zhao Z., Zhang Y., Duan B. (2018). An improved kernelized correlation filter algorithm for underwater target tracking. Appl. Sci..

[11] Zhao Q., Yang Z., Tao H. (2008). Differential earth mover’s distance with its applications to visual tracking. IEEE Trans. Pattern Anal. Mach. Intell..

[12] Dalal N., Triggs B. Histograms of oriented gradients for human detection. Proceedings of the 2005 IEEE Computer Society Conference on Computer Vision and Pattern Recognition (CVPR’05).

[13] Van De Weijer J., Schmid C., Verbeek J., Larlus D. (2009). Learning color names for real-world applications. IEEE Trans. Image Process..

[14] Simonyan K., Zisserman A. (2014). Very deep convolutional networks for large-scale image recognition. arXiv.

[15] Ma C., Huang J.-B., Yang X., Yang M.-H. (2018). Robust visual tracking via hierarchical convolutional features. IEEE Trans. Pattern Anal. Mach. Intell..

[16] Deng J., Dong W., Socher R., Li L.-J., Li K., Fei-Fei L. Imagenet: A large-scale hierarchical image database. Proceedings of the 2009 IEEE Conference on Computer Vision and Pattern Recognition.

[17] Bolme D.S., Beveridge J.R., Draper B.A., Lui Y.M. Visual object tracking using adaptive correlation filters. Proceedings of the 2010 IEEE Computer Society Conference on Computer Vision and Pattern Recognition.

[18] Henriques J.F., Caseiro R., Martins P., Batista J. (2012). Exploiting the circulant structure of tracking-by-detection with kernels. Computer Vision—ECCV 2012, Proceedings of the European Conference on Computer Vision, Florence, Italy, 7–13 October 2012.

[19] Voigtlaender P., Luiten J., Torr P.H., Leibe B. Siam R-CNN: Visual tracking by re-detection. Proceedings of the IEEE/CVF Conference on Computer Vision and Pattern Recognition.

[20] Henriques J.F., Caseiro R., Martins P., Batista J. (2014). High-speed tracking with kernelized correlation filters. IEEE Trans. Pattern Anal. Mach. Intell..

[21] Danelljan M., Häger G., Khan F., Felsberg M. (2014). Accurate scale estimation for robust visual tracking. Proceedings of the British Machine Vision Conference.

[22] Li Y., Zhu J. (2014). A scale adaptive kernel correlation filter tracker with feature integration. Computer Vision—ECCV 2014, Proceedings of the European Conference on Computer Vision, Zurich, Switzerland, 6–12 September 2014.

[23] Danelljan M., Hager G., Shahbaz Khan F., Felsberg M. Learning spatially regularized correlation filters for visual tracking. Proceedings of the IEEE International Conference on Computer Vision.

[24] Kiani Galoogahi H., Sim T., Lucey S. Correlation filters with limited boundaries. Proceedings of the IEEE Conference on Computer Vision and Pattern Recognition.

[25] Ma C., Yang X., Zhang C., Yang M.-H. Long-term correlation tracking. Proceedings of the IEEE Conference on Computer Vision and Pattern Recognition.

[26] Bertinetto L., Valmadre J., Golodetz S., Miksik O., Torr P.H. Staple: Complementary learners for real-time tracking. Proceedings of the IEEE Conference on Computer Vision and Pattern Recognition.

[27] Lukezic A., Vojir T., Cehovin Zajc L., Matas J., Kristan M. Discriminative correlation filter with channel and spatial reliability. Proceedings of the IEEE Conference on Computer Vision and Pattern Recognition.

[28] Kiani Galoogahi H., Fagg A., Lucey S. Learning background-aware correlation filters for visual tracking. Proceedings of the IEEE International Conference on Computer Vision.

[29] Mueller M., Smith N., Ghanem B. Context-aware correlation filter tracking. Proceedings of the IEEE Conference on Computer Vision and Pattern Recognition.

[30] Cao Y., Ji H., Zhang W., Xue F. (2018). Learning spatio-temporal context via hierarchical features for visual tracking. Signal Process. Image Commun..

[31] Fan J., Xu W., Wu Y., Gong Y. (2010). Human tracking using convolutional neural networks. IEEE Trans. Neural Netw..

[32] Wang N., Yeung D.Y. (2013). Learning a deep compact image representation for visual tracking. Adv. Neural Inf. Process. Syst..

[33] Wang L., Liu T., Wang G., Chan K.L., Yang Q. (2015). Video tracking using learned hierarchical features. IEEE Trans. Image Process..

[34] Li H., Li Y., Porikli F. (2015). Deeptrack: Learning discriminative feature representations online for robust visual tracking. IEEE Trans. Image Process..

[35] Zhang K., Liu Q., Wu Y., Yang M.-H. (2015). Robust visual tracking via convolutional networks. arXiv.

[36] Possegger H., Mauthner T., Bischof H. In defense of color-based model-free tracking. Proceedings of the IEEE Conference on Computer Vision and Pattern Recognition.

[37] Spokoiny V., Dickhaus T. (2015). Regression Estimation. Basics of Modern Mathematical Statistics.

[38] Rubinstein R.Y., Kroese D.P. (2016). Simulation and the Monte Carlo Method.

[39] Wu Y., Lim J., Yang M.H. (2015). Object Tracking Benchmark. IEEE Trans. Pattern Anal. Mach. Intell..

[40] Wu Y., Lim J., Yang M.-H. Online object tracking: A benchmark. Proceedings of the IEEE Conference on Computer Vision and Pattern Recognition.

[41] Jia X., Lu H., Yang M.-H. Visual tracking via adaptive structural local sparse appearance model. Proceedings of the 2012 IEEE Conference on Computer Vision and Pattern Recognition.

[42] Ross D.A., Lim J., Lin R.-S., Yang M.-H. (2008). Incremental learning for robust visual tracking. Int. J. Comput. Vis..

[43] Hong Z., Chen Z., Wang C., Mei X., Prokhorov D., Tao D. Multi-store tracker (muster): A cognitive psychology inspired approach to object tracking. Proceedings of the IEEE Conference on Computer Vision and Pattern Recognition.

